# Assessment of aortic to peripheral vascular stiffness and gradient by segmented upper limb PWV in healthy and hypertensive individuals

**DOI:** 10.1038/s41598-023-46932-0

**Published:** 2023-11-13

**Authors:** Jue Wang, Congcong Jing, Xiaojuan Hu, Ji Cui, Qingfeng Tang, Liping Tu, Shiju Zhao, Jinlian Huang, Dandan Guo, Yongzhi Li, Jiatuo Xu

**Affiliations:** 1 School of Traditional Chinese Medicine, Shanghai University of Traditional Chinese Medicine, No. 1200, Cailun Road, Pudong New District, Shanghai, 201203 China; 2 Department of Endocrinology, Seventh People’s Hospital of Shanghai, Shanghai, China; 3 Shanghai Collaborative Innovation Center of TCM Health Services, Shanghai University of Traditional Chinese Medicine, Shanghai, China; 4 The University Key Laboratory of Intelligent Perception and Computing of Anhui Province, Anqing Normal University, Anqing, China; 5 China Astronaut Research and Training Center, Astronaut Health Center Laboratory, No. 26, Beiqing Road, Haidian District, Beijing, 100094 China

**Keywords:** Cardiovascular diseases, Medical research, Diagnosis

## Abstract

Theoretically pulse wave velocity (PWV) is obtained by calculating the distance between two waveform probes divided by the time difference, and PWV ratio is used to assess the arterial stiffness gradient (SG) from proximal to distal. The aim was to investigate segmental upper-limb PWV (ulPWV) differences and the effects of hypertension and or aging on each ulPWV and SG. The study collected multi-waveform signals and conduction distances from 167 healthy individuals and 92 hypertensive patients. The results showed significant differences between ulPWVs (*P* < 0.001), with increased and then decreased vascular stiffness along the proximal transmission to the distal peripheral artery and then to the finger. Adjusted for age and sex, ulPWVs in hypertension exceeded that of healthy individuals, with significant differences between groups aged ≥ 50 years (*P* < 0.05). The hrPWV/rfPWV (heart-radial/radial-finger) was reduced in hypertension and differed significantly between the aged ≥ 50 years (*P* = 0.015); the ratio of baPWV (brachial-ankle) to ulPWV differed significantly between groups (*P* < 0.05). Hypertension affected the consistency of rfPWV with hfPWV (heart-finger). The findings suggest that segmented ulPWV is instrumental in providing stiffness corresponding to the physiological structure of the vessel. The superimposition of hypertension and or aging exacerbates peripheral arterial stiffness, as well as alteration in stiffness gradient.

## Introduction

Increased vascular stiffness is strongly associated with the onset and progression of cardiovascular (CVD) events, and central aorta such as cfPWV (carotid-femoral PWV) and baPWV (brachial-ankle PWV) can be used for risk prediction and prognostic assessment of cardiovascular diseases such as hypertension, coronary artery disease and stroke^1–3^. It has been shown that changes in vessel elasticity precede changes in vessel stenosis^4^. Therefore the PWV technique for non-invasive assessment of vascular stiffness has been a hot topic of clinical research and application since its introduction. In recent years, the assessment of local peripheral vascular stiffness and its role in the arterial stiffness gradient (SG) has gained importance with the deepening of machine studies and the advancement of equipment technologies^5,6^. Physiological SG formation is due to the naturally uneven distribution of elasticity in arteries throughout the body. As the distance from the heart increases, the elastin content of the arterial structure decreases, the collagen fiber content increases, and the diameter decreases, resulting in increased stiffness of the aorta to the peripheral arteries. The reduction, disappearance or even reversal of SG is thought to lead to hemodynamic damage in hyper perfused organs such as the brain and kidneys^7,8^. Fortier et al.^7^ applied the ratio of cfPWV to crPWV (carotid-radial PWV) to the prognosis of dialysis patients, with the results supporting that the PWV ratio is a better predictor of dialysis mortality than cfPWV. Additional studies support the aortic-peripheral PWV ratio as an independent indicator for evaluating vascular ageing and cardiovascular disease risk^9,10^. Although there are opposing viewpoints, the quantification of peripheral vascular stiffness is gradually highlighting its value^11–13^.The ulPWV contains information on vascular stiffness from the heart to the axillary, brachial and radial arteries as well as to the terminal circulation of the fingertips. The diameter of blood vessels gradually decreases during the process. For example, the average internal diameter of the brachial artery is approximately greater than 4 mm to that of the small arterial conduit, which averages less than 2.3 mm^14,15^. Changes in the composition of the vessel wall and the diameter cause changes in the elasticity of the vessel. Compared to the aorta and lower limb arteries, ulPWV is easier to measure and portable^7,16^, however there are fewer studies of segmented ulPWV. Upper limb arteries can be used to evaluate the efficacy of clinical pharmacological interventions in hypertension^17,18^ and also to study the effects of exercise or lifestyle on vascular stiffness^19–21^, all of which place demands on the methods and data of local peripheral arterial PWV. Although baPWV is widely used in Asia, the accuracy of this index is affected by diseases such as stenosis of the upper limb arteries, and it has also been suggested that baPWV combines the information on peripheral arterial stiffness^8,22^. Thus, we propose that there is a value in using PWV to assess upper limb arterial stiffness.

The current study found that higher baseline aortic stiffness is associated with reduced stiffness in muscular conduit arteries, and the relationship affects the circulatory system in a two-sided manner^7^. Age does not affect peripheral arterial stiffness as much, but always plays a role in mixing with disease^8,23^. Hypertension is a strong independent predictor of risk of cardiovascular events, and since the smaller diameter arteries in the resistance vessels have a key role in controlling systemic blood pressure, alterations in vascular remodeling, particularly in small arteries, are strongly associated with hypertensive disease progression and severity^24,25^. Large artery elasticity due to hypertension is thought to be similar to the results of aging^26,27^, yet there are fewer PWV studies focusing on the effects of hypertension combined with age factors on peripheral resistance and microvessels. The present study points out that hfPWV (heart-finger PWV), i.e., complete ulPWV, combines the elasticity of the central aorta, middle arteries, small arteries and microvessels. Considering the different compositional ratios of the arterial wall components, such as the content of smooth muscle, collagen fibers and elastic fibers^8^. We hypothesized that the degree of stiffness in the different segments of the arteries of the upper limbs is not uniform. Therefore, we obtained different waveform data for the upper limb using three signals: electrocardiogram, pressure wave and light volume wave, followed by calculating six ulPWVs based on distance divided by time. Firstly, the differential characteristics of different ulPWV of the upper limb in healthy subjects and their influencing factors were studied. Then, the differences in each ulPWV and SG (PWV ratio) between hypertensive patients and healthy subjects in two age groups were investigated. The SGs of this study were derived from different ulPWVs and ulPWV versus baPWV, the former exploratory study of stiffness gradients during arterial conduction in the upper limb with the brachial, radial arteries as the nodes; whereas the latter may be more focused on the differences in the stiffness of arteries in the upper and lower reaches of the body. Finally different ulPWV consistency analyses resulted in representative indicators.

The aims of this paper are (1) to analyze the differences in ulPWVs and the influencing factors (age, blood pressure and heart rate) in the context of the structure of the corresponding vessels; and (2) to explore the variability of ulPWVs and the associated SGs under the influence of synergistic hypertension and or aging. Both for proposing a segmented approach to the study of ulPWVs and explaining its necessity.

## Materials and methods

### Study population

This was a cross-sectional study that included a total of 167 healthy subjects (84 females) and 92 hypertension patients (25 females) after excluding data with missing or abnormal waveforms. Basic information on all subjects is shown in Table 1, and all subjects and data were obtained from the physical examination center and cardiovascular department of Shu Guang Hospital, affliated to Shanghai University of Traditional Chinese Medicine. The Medical Ethics Committee of the Shu Guang Hospital affliated to Shanghai University of Traditional Chinese Medicine approved the study (Approval Numerber: 2018-626-55-01), and informed consent was obtained from all included subjects according to the Declaration of Helsinki. All participants had complete data on segmented ulPWV and baPWV, as well as the related clinical indicator data. The health inclusion criteria were age between 18 and 65 years, and without hypertension, cardiovascular disease, diabetes and chronic kidney disease, besides medication taking were also excluded. Hypertension was defined on (i) previous diagnosis of hypertension, (ii) systolic blood pressure ≥ 140 mmHg or systolic blood pressure ≥ 90 mmHg. Due to LEAD (lower extremity artery disease) can affect the accuracy of PWV measurements^20^, so that ABI (ankle-brachial index) < 0.9 or > 1.4 was the exclusion criteria for all participants.

**Table 1:** Clinical and hemodynamic characteristic of subjects in the study (${\overline{\text{x}}} \pm {\text{SD}}$).

Subjects (n)	Age (yr.)	Male (n, %)	Height (cm)	BMI (kg/m^2^)	SBP (mmHg)	DBP (mmHg)	Heart rate (beats/min)
Health (167)	39.51 ± 10.07	83 (49.70)	166.12 ± 8.21	23.39 ± 2.95	116.88 ± 10.78	70.79 ± 9.07	69.01 ± 9.14
Hypertension (92)	52.12 ± 0.45	67 (72.83)	166.98 ± 8.07	25.22 ± 2.76	145.90 ± 15.35	89.34 ± 10.44	75.08 ± 12.06

### Clinical data collection

Baseline clinical data of participants were obtained (i.e., age gender, height, weight) when them accepted hemodynamic evaluation, besides, hypertension patients were also inquired duration of hypertension, history of hypotensive drugs taking. BMI (body mass index) was calculated as weight divided by height in square meters. Hemodynamic indicator such as baPWV, blood pressure and heart rate were obtained using an atherosclerosis diagnostic device (OMRON, BP-203RPEIII, Japan), which provided bilateral side baPWV measured from the cuffs wrapped on both brachial arms and ankles. Hemodynamic indicators were measured in a quiet test room with similar temperature after 10 min of rest in the supine position.

### UlPWV measurement and analysis

**Figure 1:**
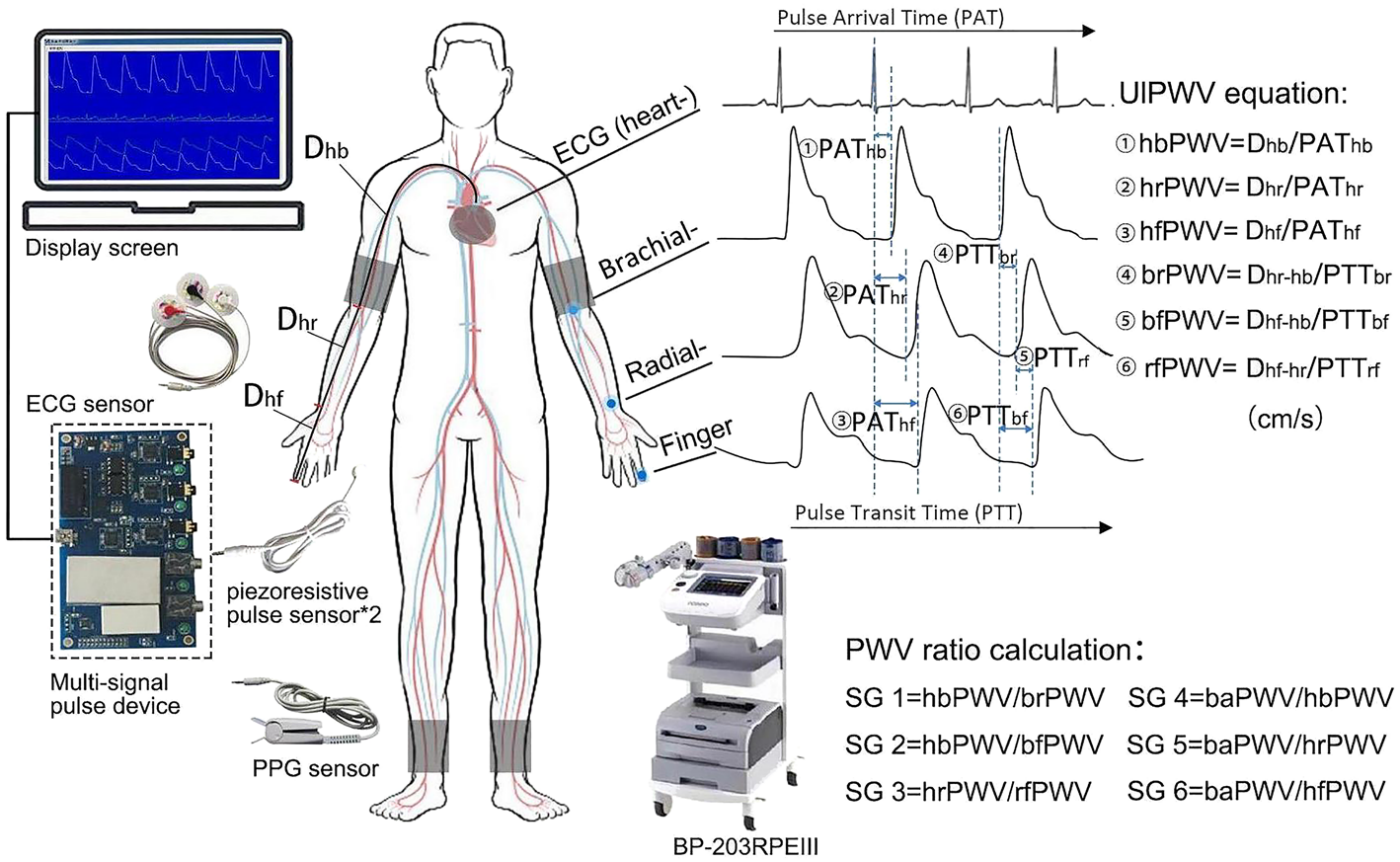
Demonstration of PWV waveform acquisition and calculation approach for ulPWV and PWV ratio. *Note*: All four waveform signals of the heart-upper limb on the figure were collected by the Multi-signal pulse device, with the sampling probes as blue dots. BaPWV was collected by the BP-203RPEIII, whose taping was positioned as grey blocks.

This study collected waveform signals from different sites of the aorta-upper limb using the multi-channel signal pulse device. The device has an automatic sampling frequency of 200 Hz, and USB full-duplex serial communication interface mode; it is also equipped with five signal interfaces, which are three pulse acquisition channels, one ECG channel, and one volumetric waveform channel. Combined with piezoresistive pulse sensor, leadwire and volumetric waveform finger clip to enable simultaneous acquisition of pulse wave, ECG and finger volumetric waveform signals. Figure 1 shows the acquisition diagram and display interface, which requires multiple waveforms to be stable at the same time before data is acquired for at least half a minute. The ECG-R wave is used as the start marker and the pulse wave reaches the brachial, radial and finger end as PAT heart-brachial, hear-radial and heart-finger respectively; while the pulse wave at the brachial, radial and finger end is spaced as PTT brachial-radial, brachial-finger and radial-finger respectively, with different waveform feature extraction algorithms for PAT and PTT. In addition, the participants extended their arms and torso at approximately 90° in the standing position, and the actual acquisition needs to minimize the interference of clothing, using a tape measure to measure the transmission distance separately. The 3rd intercostal space (3I.S.) as the starting position of the heartbeat, and measured the distance to the suprasternal notch^28^; then measured the distances from the suprasternal notch to the brachial artery, radial artery, and fingertip as the endpoints, respectively.

We used MATLAB 2016 software (MathWorks, Natick, MA) for multiple waveform processing and feature point extraction. Firstly, the multiple waveforms were manually filtered and de-noised for analysis. Then, the peak method was used for ECG-R waveform identification, while the remaining three waveforms used the second derivative maximum and the intersecting tangent to cooperate in identifying the waveform “onset point”. The PAT was taken as the lesser of the two methods, the PTT was taken as the mean of the two methods, and the manual monitoring was done across all multiple waveforms for feature point identification. We also attempted to use the peak or minimum method, but found that the valleys of the actual waveforms were offset and noisy, and that the peak method lost much of the waveform information^5^, so neither was adopted. UlPWV is calculated by dividing the body surface conduction distance (D) corresponding to the multiple waveforms by the pulse arrival time (PAT) or pulse transit time (PTT)^5^. Each ulPWV, as well as baPWV, was obtained using the mean value of the two-sided measurements^29,30^. The six ulPWVs corresponding to different arterial segments were hbPWV (heart-brachial PWV), hrPWV (heart-radial PWV), hfPWV (heart-finger PWV), brPWV (brachial-radial PWV), bfPWV (brachial-finger PWV), and rfPWV (radial-finger PWV), in cm/s in accordance with baPWV (as shown in Fig. 1).

### Arterial stiffness gradients (PWV ratios) calculation

The energy changes in human haemodynamics transmitted from the aorta to the peripheral arteries are mainly presented through the arterial stiffness gradient, and PWV ratio between the two arterial segments is usually calculated to obtain the SG index^7^. In this study, ulPWV-related SG was considered to contain some aortic stiffness information, combined with the ratio relationship with the local PWV of the neighboring upper limb, which were hbPWV divided by brPWV, hbPWV divided by bfPWV and hrPWV divided by rfPWV, respectively; baPWV, although it contains some peripheral arterial stiffness, it is undeniable that it is mainly aortic stiffness related. Therefore it was taken as the numerator and divided by the three ECG-related ulPWVs (hbPWV, hrPWV and hfPWV), respectively, and the calculation of the specific six SGs were shown in Fig. 1. All data was acquired by two researchers using the same techniques and equipment.

### Statistical analysis

All values are expressed as mean ± SD. One-way ANOVA was used to compare ulPWV in healthy individuals of different age groups, all with Bonferroni correction. Differences between hfPWV and other ulPWV as well as baPWV were compared separately using paired samples *t*-tests. One-way ANOVA was used to compare ulPWV in healthy individuals in different age groups, all using Bonferroni correction. We used Pearson correlation coefficients to analyze the bivariate correlations between ulPWV and the influencing factors as well as the different PWV, and the Bland–Altman method to analyze the consistency of ulPWV. Independent samples *t*-tests were used to compare PWV across gender in healthy individuals, as well as PWV and PWV ratio in healthy individuals with hypertension in two age groups. Healthy individuals were matched for age and sex for hypertension using propensity scores, and analyses were performed using SPSS statistical package version 26 (SPSS Inc., Chicago), *P* value less than 0.05 was considered as statistically significant.

## Results

**Figure 2:**
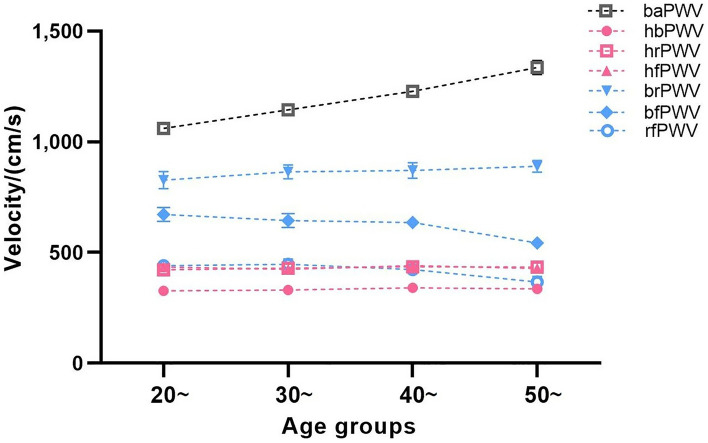
Trend in ulPWVs and baPWV of healthy participants at age groups.

**Figure 3:**
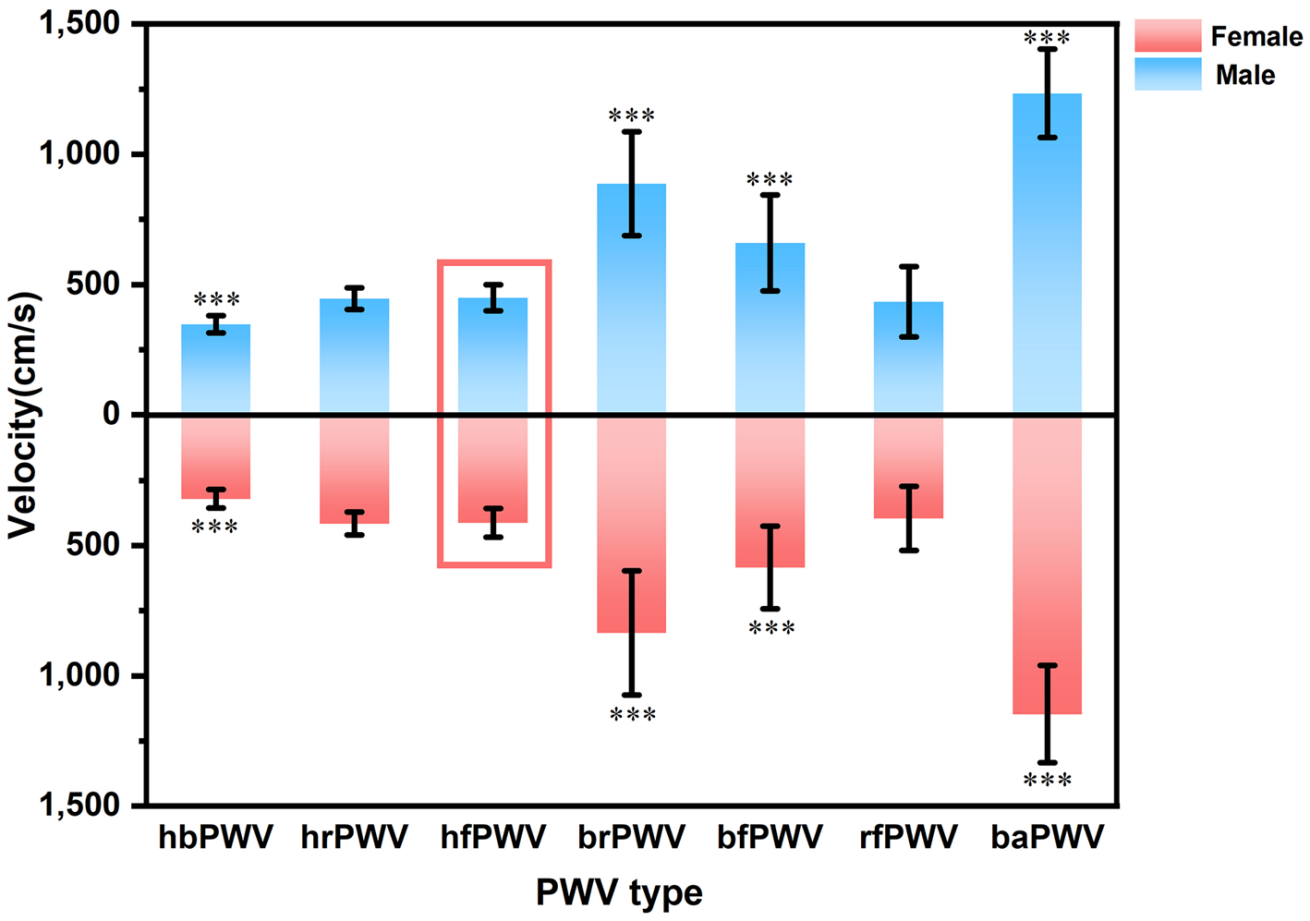
Differential characteristics of ulPWVs and baPWV in healthy men and women. *Note*: Differential results are labelled from the comparison of different PWVs with hfPWV, *** means *P* < 0.001, and differentiate between men and women in the form above and below.

Table 1 presents basic information on the clinical characteristics and hemodynamics of the sample of healthy and hypertensive subjects in both groups. Stratified by 10 years of age, there was no difference in the proportion of males and females in each of the groups listed in Table 2 (*P* = 0.407), and the results of the cross-sectional and longitudinal comparisons of the ulPWV in each group are also shown. Comparison of results between age groups showed no significant difference in ulPWV except for bfPWV, which was statistically different (*P* = 0.027); significant differences were found for all ulPWVs in the same age group (all *P* < 0.001). Figure 2 demonstrates the trend of different ulPWV with age groups in healthy individuals, with baPWV added as a reference. It can be seen that baPWV was the largest in all age groups, and its difference with hbPWV was the largest and with brPWV was the smallest, and it also showed an increasing trend with age. However, changes in ulPWV were relatively smooth between age groups, suggesting that small segments of the aorta and peripheral arteries upstream of healthy individuals were less affected by age, and the trend was even towards a decrease in ulPWV with increasing age. Figure 3 distinguishes healthy individuals between males and females plotting the differential characteristics of different ulPWVs, with similar trends in ulPWVs for males and females, and significant differences between hbPWV and hfPWV (*P* < 0.001) due to the incorporation of small and medium muscle arteriolar stiffness leading to an increase in hrPWV and hfPWV. When upper limb arteries were observed in segments, brPWV, which consistently corresponded to small arteries in forearm muscles, was the largest, and bfPWV, which combined forearm as well as radial finger segments, was the second largest, with statistically significant differences in both compared to hfPWV (*P* < 0.001); whereas rfPWV corresponding to radial-finger decreased, and although they corresponded to different vascular physiology, its values were not statistically different from hfPWV. Trends in hbPWV, brPWV, and rfPWV showed a first increase and then a decrease in PWV delivered along the aorta- peripheral arteries-microvascular arterial tree.

**Table 2:** Basic characteristics of healthy participants by age group of ten years.

Age group (n)	Male (n, %)	hbPWV	hrPWV	hfPWV	brPWV	bfPWV	rfPWV	*P* for ulPWV
20 ~ year (32)	15 (46.88)	326 ± 35	419 ± 43	430 ± 51	820 ± 219	669 ± 182	430 ± 105	< 0.001
30 ~ year (52)	29 (54.72)	332 ± 43	429 ± 56	424 ± 61	860 ± 229	628 ± 200	427 ± 144	< 0.001
40 ~ year (51)	21 (41.18)	341 ± 34	436 ± 41	441 ± 52	871 ± 249	635 ± 164	423 ± 118	< 0.001
50 ~ year (32)	18 (56.25)	335 ± 31	435 ± 35	428 ± 56	890 ± 154	544 ± 114	367 ± 141	< 0.001
Total (167)	84 (49.41)	334 ± 37	430 ± 46	431 ± 56	861 ± 221	622 ± 175	415 ± 130	< 0.001
*P*	0.407	0.329	0.398	0.467	0.630	0.027	0.138	–

**Figure 4:**
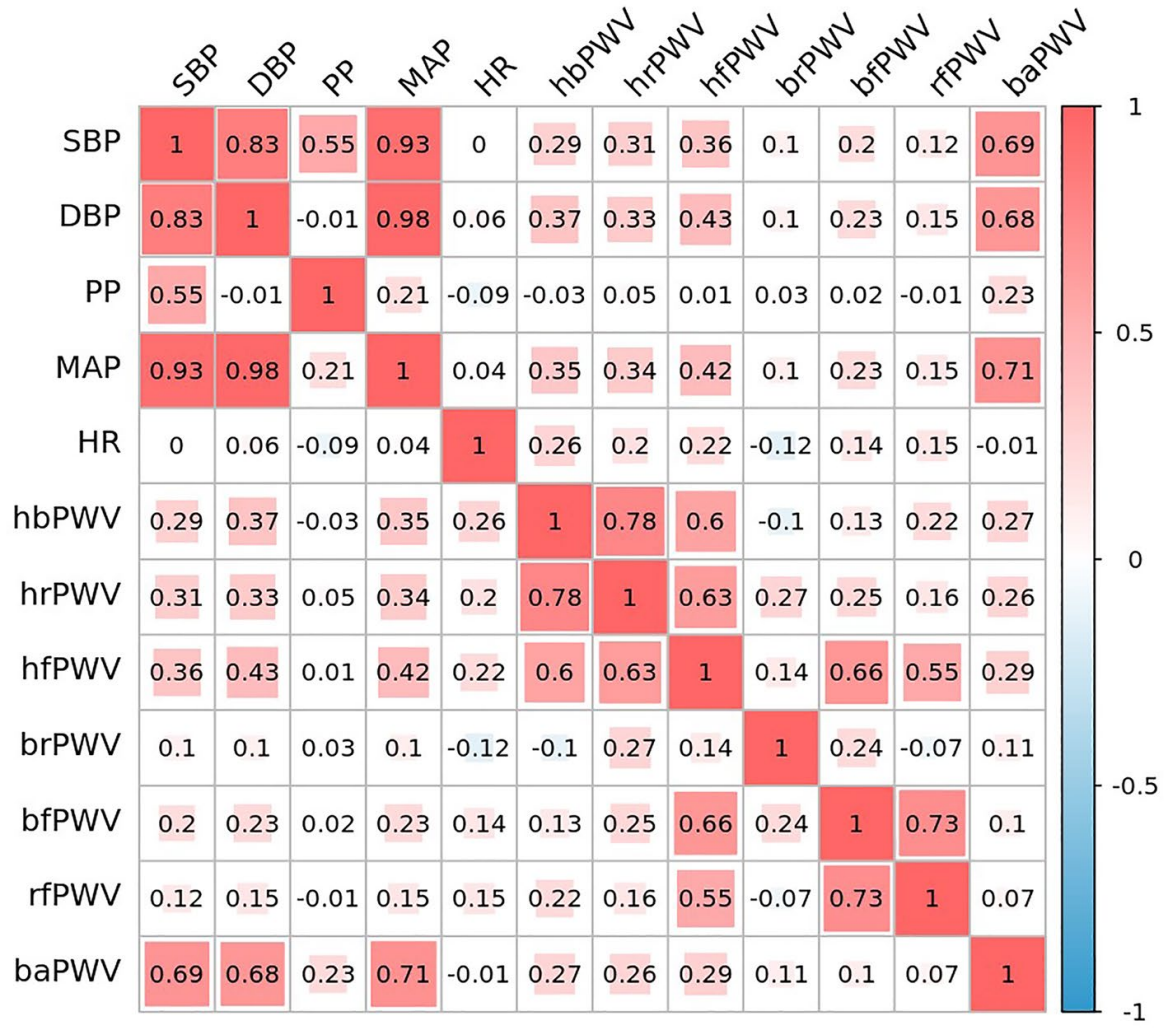
Correlation study of ulPWVs and baPWV with blood pressure and heart rate.

Figure 4 shows bivariate correlations of ulPWV and baPWV with blood pressure and heart rate indices, which showed that baPWV had the highest correlation with blood pressure indices (SBP, DBP and MAP) (R = 0.69, R = 0.68 and R = 0.71, respectively, *P* < 0.01). Upper limb hbPWV, hrPWV and hfPWV also provided evidence of moderate correlation with blood pressure, while the remaining ulPWVs were weakly correlated with blood pressure. Heart rate correlated only with ulPWV and not with baPWV. There were different degrees of correlation between different PWVs. Three ulPWVs related ECG were correlated with baPWV (R = 0.27, R = 0.26 and R = 0.29, respectively, all *P* < 0.01); them were also strongly correlated with each other; hfPWV was strongly correlated with bfPWV (R = 0.66, *P* < 0.01) and moderately correlated with rfPWV (R = 0.55, *P* < 0.01), and bfPWV was strongly correlated with rfPWV (R = 0.73, *P* < 0.01), which may be related to the specificity of the microvessels of the radial-finger.

**Figure 5:**
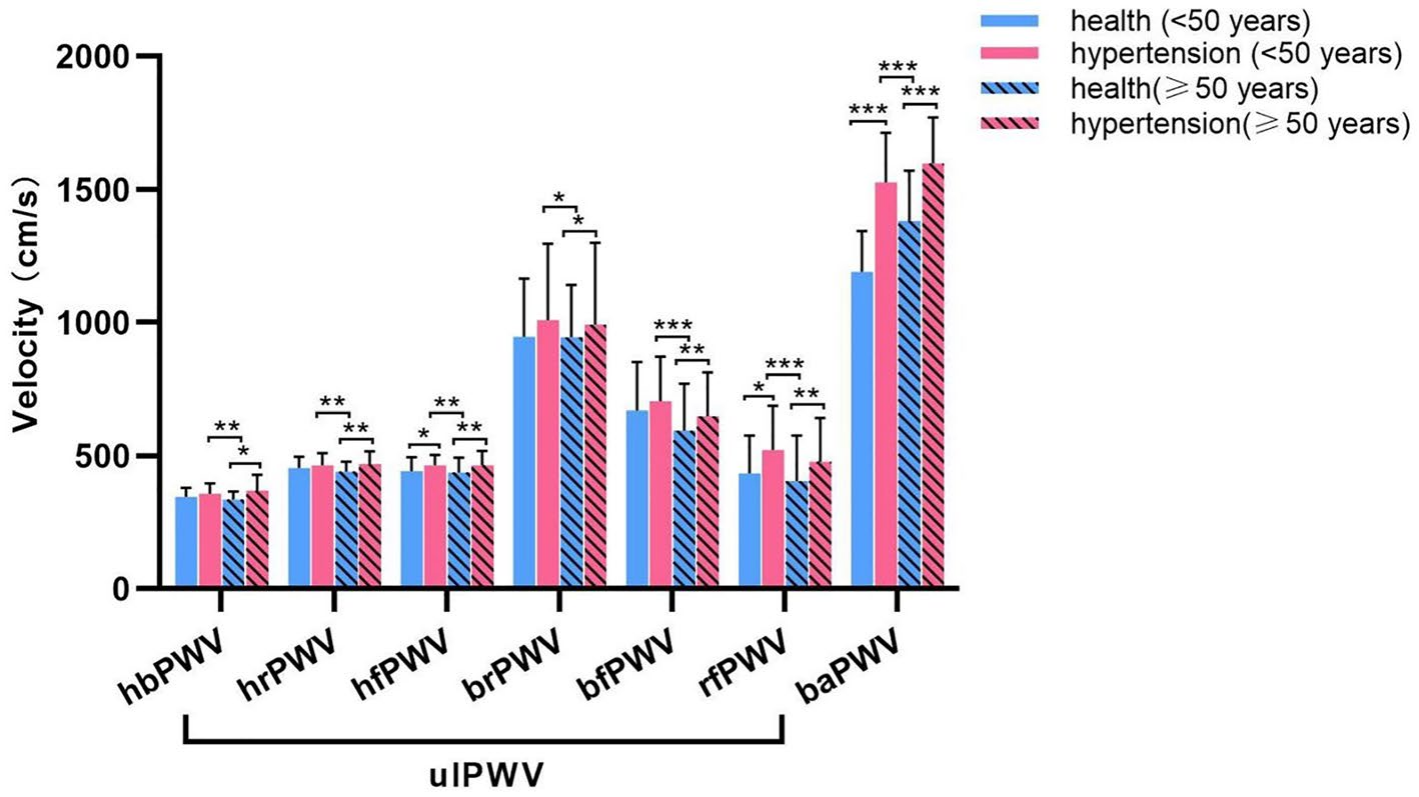
Comparison of ulPWVs and baPWV in the health and hypertensive patients with age < 50 years and age ≥ 50 years.

Based on the results of ulPWV comparisons among healthy individuals of different ages and sexes, and considering the age profile of hypertensive patients, the present study was conducted using the age of 50 years as the cut-off, and propensity scores were used to match sex and age. The basic characteristics of healthy individuals and hypertensive patients in both age groups (age < 50 years and age ≥ 50 years) are shown in Table 3, and there were no statistically significant differences in their age and sex group comparisons. Figure 5 shows the comparative results of ulPWV and baPWV for healthy individuals and hypertensive patients of both age groups respectively. Regardless of age group, each ulPWV and baPWV of hypertensive patients exceeded those of healthy individuals, suggesting that hypertension leads to increased stiffness of the aorta and different arteries of the peripheral circulation in the human body compared to healthy individuals. There were statistically significant differences in hfPWV, rfPWV and baPWV in hypertensive patients aged < 50 years compared with healthy individuals of the same age (*P* = 0.038, *P* = 0.011 and *P* < 0.001, respectively); when aged ≥ 50 years, there were statistically significant differences in each of the ulPWVs in hypertensive patients compared with healthy individuals of the same age (hbPWV, *P* = 0.014; hrPWV, *P* = 0.001; hfPWV, *P* = 0.005; brPWV, *P* = 0.019; bfPWV, *P* = 0.001; rfPWV, *P* = 0.002; baPWV, *P* < 0.001), suggesting that the stiffening of all segments of the upper limb arteries was more pronounced in hypertensive patients of this age group compared with healthy individuals of the similar age. It was also observed that each ulPWV and baPWV was increased in hypertensive patients aged < 50 years (mean age 39.37 ± 6.61 years) compared with healthy people aged ≥ 50 years (mean age 53.78 ± 2.29 years), and the differences were statistically significant (hbPWV, *P* = 0.008; hrPWV, *P* = 0.004; hfPWV, *P* = 0.002; brPWV, *P* = 0.042; bfPWV, *P* < 0.001; rfPWV, *P* < 0.001; baPWV, *P* < 0.001), suggesting that hypertension in combination with aging leads to a more prevalent and pronounced stiffening of arteries in all segments of the upper limbs in patients than in healthy individuals of the same age; hypertension and aging may be underlying different biological expressions that affect arterial stiffness. Table 4 shows an exploratory analysis of the relationship between ulPWV related ratios as well as baPWV to ulPWV in hypertensive and healthy individuals at two age levels according to the above. There were no statistically differences between the groups of SG 1 and SG 2 based on hbPWV, brPWV and bfPWV, but the values were all less than one; while hrPWV/rfPWV were all greater than one, the hypertensive patients in both age groups were lower than the healthy individuals, and the difference in the hypertensive patients with aged ≥ 50 years compared with the healthy individuals was significant (*P* = 0.015). This suggests that the superimposition of hypertension and aging leads to a reduction in the stiffness gradient of muscular medium and small arterial conduction to the radial finger segments. All three SGs of baPWV divided by ulPWV were greater than one and increased in hypertensive patients compared with healthy individuals, with all differences between groups significant (*P* all < 0.001 for age < 50 years; *P* = 0.007, *P* = 0.002, and *P* = 0.032 for age ≥ 50 years). This suggests that the effects of hypertension resulted in increased baPWV and each ulPWV at both ages, but the increase in brachial-ankle artery stiffness was greater than that of the upper-limb arteries.

**Table 3:** Characteristics of study population.

Group (n)	Male (%)	Age (years)	BMI (kg/m^2^)	SBP (mmHg)	DBP (mmHg)	Heart rate (beats/min)
Age < 50 years						
Health (n = 45)	31 (68.89)	39.09 ± 6.56	23.59 ± 2.77	118.78 ± 10.77	72.29 ± 8.62	67.96 ± 8.01
Hypertension (n = 35)	25 (71.42)	39.37 ± 6.61	25.24 ± 3.17	147.33 ± 14.30	94.47 ± 10.58	77.29 ± 10.22
* t*/*x*^2^	0.254	− 0.19	− 2.484	− 10.186	− 10.331	− 4.578
* P*	0.614	0.849	0.015	< 0.001	< 0.001	< 0.001
Age ≥ 50 years						
Health (n = 32)	18 (56.25)	53.78 ± 2.92	24.57 ± 2.62	121.09 ± 9.62	74.86 ± 7.41	64.53 ± 7.29
Hypertension (n = 36)	22 (61.11)	51.94 ± 5.38	25.03 ± 1.95	149.15 ± 10.72	92.00 ± 8.34	73.47 ± 14.55
* t*/*x*^2^	0.165	1.72	− 0.828	− 11.305	− 8.909	− 3.142
* P*	0.684	0.09	0.411	< 0.001	< 0.001	0.003

**Table 4:** Comparison of SGs (PWV ratios) in the health and hypertensive patients on two age groups.

SG (PWV ratio)	< 50 years			≥ 50 years		
	Health	Hypertension	*P*	Health	Hypertension	*P*
SG 1 (hbPWV/brPWV)	0.39 ± 0.10	0.39 ± 0.15	0.896	0.39 ± 0.09	0.39 ± 0.16	0.975
SG 2 (hbPWV/bfPWV)	0.56 ± 0.16	0.54 ± 0.14	0.645	0.64 ± 0.14	0.57 ± 0.19	0.108
SG 3 (hrPWV/rfPWV)	1.17 ± 0.43	1.00 ± 0.42	0.076	1.33 ± 0.45	1.07 ± 0.41	0.015
SG 4 (baPWV/hbPWV)	3.47 ± 0.55	4.29 ± 0.62	< 0.001	4.01 ± 0.61	4.44 ± 0.65	0.007
SG 5 (baPWV/hrPWV)	2.63 ± 0.40	3.31 ± 0.47	< 0.001	3.09 ± 0.44	3.41 ± 0.40	0.002
SG 6 (baPWV/hfPWV)	2.71 ± 0.39	3.29 ± 0.39	< 0.001	3.17 ± 0.54	3.44 ± 0.48	0.032

**Figure 6:**
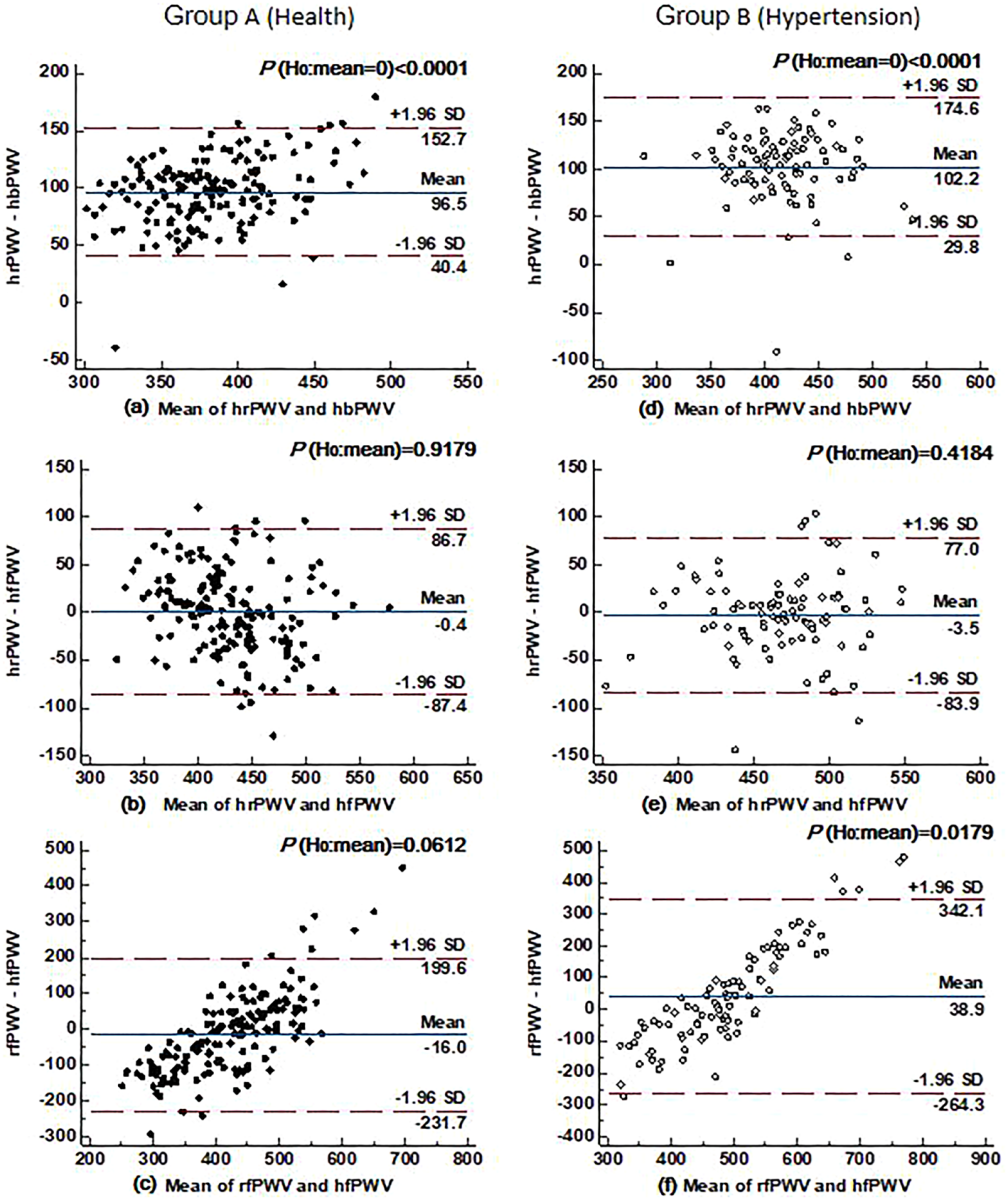
Agreement between hrPWV and hbPWV, hrPWV and hfPWV, as well as rfPWV and hfPWV in healthy (n = 167) and hypertensive (n = 92) individuals. *Note*: the Bland–Altman plot is the difference between the two ulPWVs, the red horizontal dashed line indicates the upper and lower limits of the 95% consistency bounds, i.e., 1.96 times the standard deviation; the blue horizontal solid line in the middle represents the mean of the difference, and the P indicates the assumption that the mean of the difference is zero, with < 0.05 being considered significant.

Figure 6 shows the concordance analysis of ulPWV in all healthy individuals and hypertensive patients. It can be seen that the agreement between hrPWV and hbPWV was poor in both groups (all *P* < 0.0001), and the agreement between hrPWV and hfPWV was better in both groups (*P* = 0.9179 and *P* = 0.4184, respectively), suggesting that hrPWV can represent hfPWV to some extent, but not hbPWV. In line with the previous Fig. 4 bivariate agreement between the ulPWV in each ulPWV correlation results, the agreement between rfPWV and hfPWV was high in healthy individuals (*P* = 0.0612), but poorer in hypertensive individuals (*P* = 0.0179), suggesting that the terminal microvessels corresponding to rfPWV need to be evaluated separately.

## Discussion

This study demonstrates that multiple ulPWVs can be derived to segment the characteristics of the arterial stiffness index of the upper extremity by calculating the conduction times from the heart to the brachial artery, radial artery, and fingertip, respectively. It was found that (1) the vascular stiffness assessed by ulPWV correlates with the physiological structure of the elastic aorta, large and small muscular arteries, or microvessels counterparts; (2) UlPWV is less affected by age than baPWV, and there are differences in correlation of the two types of PWVs with blood pressure and heart rate; (3) UlPWVs and associated SGs can be used as an assessment of changes in vascular stiffening and stiffness gradients in upper limb arteries affected by hypertension and or aging.

Aortic PWV is recognized as a reliable assessor and predictor of cardiovascular events and mortality, but the lack of a clear relationship between peripheral arterial stiffness and cardiovascular outcomes. This may obscure the characteristics of the stiffness of small arteries in the peripheral muscles and the physiological role that they play in the regulation of the pressure of the circulatory pulsation^31,32^. This study firstly focuses on the PWV of different segments of arteries in the upper limb of healthy individuals, and all ulPWVs are smaller than the combined stiffness of the aorta and peripheral arteries, baPWV, and change more steadily with age. This suggests that arteries in the upstream of the human body are less stiff than those in the downstream, which is in line with the fact that the lower limbs of the body are subjected to higher hydrostatic pressures than those of the upper limbs^33^. However ulPWV even tended to decrease with age, a paradox that has also been confirmed in other studies^23,34,35^. It has been proposed that peripheral muscular arteries may be designed to match the increased stiffness of the large central arteries by enhancing their own compliance to accept the transmission of blood volumes with increased pulsatile pressure, but this alteration affects the myocardium and microcirculation in a two-sided manner^7^. Also the decrease in rfPWV and bfPWV with age may be due to the specificity of the small vessels of the microcirculation, whose values are close to those of hrPWV and hfPWV, which are easily neglected if the stiffness of the arteries of the upper limbs is assessed as a whole^11^. Kavroulaki et al.^36^ found that blood flow to the fingers was rather reduced in premenopausal women and that younger women had a tendency to peripheral vasoconstriction. The peripheral vessels of the fingertips became progressively thinner, and the spectral waveforms of the fingertip arteries were all monophasic^37,38^. This suggests that factors such as vessel structure and internal diameter need to be taken into account when assessing the stiffness of microvessels using PWV^39^. The ulPWV of the aorta-peripheral middle and small arterioles-microvessels increases and then decreases, also emphasizing that changes in the wall composition and reductions in vessel diameter from proximal to distal of the arterial tree can lead to changes in vascular elasticity^14,15^. In healthy people ulPWV is similarly affected by blood pressure, although less so than the relationship between baPWV and blood pressure. The correlation between heart rate and ulPWV is stronger than that of baPWV, and some studies have shown that "active" or natural state heart rate is associated with arterial stiffness, implying that when heart rate is chronically fast, it may lead to degenerative changes in the arteries^40^. This "circulatory fatigue" is better reflected in ulPWV, which could inform future studies on the mechanisms affecting arterial stiffness in the upper limb.

The main pathological hallmark of hypertension, a key risk factor for cardiovascular disease, is the increase in peripheral vascular resistance due to structural and functional changes in large conducting and small resistance arteries^41^. The present study firstly clarifies that hypertension causes an increase in each ulPWV and baPWV compared to healthy individuals of the same age, suggesting that hypertension affects vascular stiffness in a systemic manner. Furthermore the significance of the increase in ulPWV was more prevalent in hypertensive patients aged ≥ 50 years than in hypertensive patients aged < 50 years. This may correlate with the involvement of ulPWV in reflecting the progression of hypertension, especially in the smaller diameter arteries of the resistance vessels, whose vascular changes are highly correlated with the severity of hypertension^40,41^. In addition, the difference between hypertensive patients aged < 50 years and healthy individuals aged ≥ 50 years seemed to reflect the relationship between aging and hypertension in the context of vascular structural alterations. Su et al.^42^ found that Klotho protein cycling levels (anti-aging gene expression) were lower in nonelderly hypertensive patients than in elderly non-hypertensive individuals, which was consistent with the performance of ulPWV and baPWV in this study. It suggests that hypertension may have a greater impact on vascular structural alterations than aging, and it also suggests that ulPWV may serve as an outgrowth of vascular gene expression. Structural–functional changes in different vessels will inevitably bring about changes in SG, and examining the differences in the ratios of neighboring ulPWVs (SG 1–3) as well as the ratios of baPWV to the three ulPWVs (SG 4–6), respectively. In hypertensive and healthy individuals, it was found that the difference between hrPWV/rfPWV was significant in hypertensive patients with aged ≥ 50 years and in healthy individuals. It is suggested that hypertension combined with aging may amplify alterations in the stiffness gradient of cardiac-radial PWV conduction to radial-finger PWV, whose values decrease or even disappear indicating alterations in microcirculatory pulsatile pressures and hemodynamics thus leading to organ damage^7,43^. The absence of differences in the other ASGs does not imply that there is no change in vascular structural function, but rather suggests that hypertension causes physiological degradation of the large arteries similar to that of aging, and that vascular parallel remodeling of the muscular arteries of the upper limbs may have occurred^9,41^. Differences in vascular remodeling of conduit arteries and peripheral radial arteries under the influence of hypertension, with intima-media thickness of the latter found not to be significantly related to local pulse pressure^25,44,45^. This may explain the manifestation of hbPWV/brPWV and hbPWV/bfPWV and emphasize the heterogeneity of the microvessels at the finger tips. SG 4–6 were all > one and significantly different. This may correlate with differences in the vascular physiology of the upstream and downstream arteries, which are subjected to different pressures over time^33^; there are differences in the changes in stiffness of aortic-lower limb and upper limb arteries that are induced by high blood pressure and or ageing, and that the ratios of baPWV to ulPWV help to visualize these exacerbated differences^7,41^.

The strength of this study is the segmented study of ulPWV using multiple signals, which takes into account the different requirements of the PAT and PTT algorithms during multiple ulPWV calculations and proposes a segmented approach to measure ulPWV^7,43^. Different ulPWVs have differences that reveal, to some extent, the physiological properties of the central aorta to the peripheral vasculature that gradually stiffen due to changes in the composition of the vessel wall (decrease in elastin and increase in collagen and smooth muscle cells)^7,43^. Although rfPWV and hfPWV values are close, the underlying vascular physiology is very different. The study utilizes ulPWV to complement the stiffness information of the upper limb arteries, making it possible to evaluate the overall degree of atherosclerosis in the presence of hypertension and or aging factors. Then, it explores the changes in ulPWV-involved SGs in response to physiological and pathological factors, providing information for the discovery of potential indicators for vascular health assessment. Consistent evaluation suggests that hrPWV can be used as a proxy for upper extremity arterial stiffness, but not for hbPWV, while rfPWV deserves separate evaluation.

There are some limitations to be mentioned. Firstly, hand microcirculation is not necessarily an endpoint, and in this study rfPWV was measured only using fingertip volume wave as a relative endpoint. Furthermore, there are many and confounding factors affecting vascular stiffness, and only age, blood pressure and gender were considered in this study; it needs to be made clear that there may be interactions between these factors, and that other factors such as smoking, obesity and lifestyle deserve to be investigated in depth^46,47^. In addition, pathologies other than hypertension deserve to be explored^48^. PWV is widely used as an assessment tool for vascular stiffness, but it needs to be clear that vascular aging and arterial stiffness are not identical and that the two are in a mismatched symbiotic relationship^49^. UlPWV is expected to inform the application of ultrasound technology on a large sample basis in the future, with both working together to assess structural and or functional changes in the vascular system.

## Conclusion

In conclusion, different ulPWVs in the aorta-upper limb segment closely correlate with their corresponding vascular structures, with increasing and then decreasing stiffness along the proximal to distal peripheral arteries and then to the fingers, with specificity for rfPWV. Different ulPWV reflects the effect of hypertension and or aging factors on vascular stiffness, and the superposition of both risk factors leads to an increase in ulPWV and a more pronounced attenuation of hrPWV/rfPWV. Also ulPWV exploratively constructs a ratio to baPWV, and hypertension may cause a faster increase in the degree of combined aortic-lower limb arterial stiffness than in upper limb arteries. Although assessment of upper limb peripheral arterial stiffness is not as important as the aorta, the vascular physiology behind it and changes in the arterial stiffness gradient deserve attention.

## Data Availability

The datasets generated and/or analyzed during the current study are not publicly available due to the agreement of the funding but are available from the corresponding author on reasonable request.

## Abbreviations

PWV: Pulse wave velocity
BaPWV: Brachial-ankle pulse wave velocity
UlPWV: Upper-limb pulse wave velocity
HbPWV: Heart-brachial pulse wave velocity
HrPWV: Heart-radial pulse wave velocity
HfPWV: Heart-finger pulse wave velocity
BrPWV: Brachial-radial pulse wave velocity
BfPWV: Brachial-finger pulse wave velocity
RfPWV: Radial-finger pulse wave velocity
SG: Stiffness gradient
